# Subsequent surgery after primary ACLR results in a significantly inferior subjective outcome at a 2-year follow-up

**DOI:** 10.1007/s00167-021-06850-y

**Published:** 2021-12-31

**Authors:** Christoffer von Essen, Riccardo Cristiani, Lise Lord, Anders Stålman

**Affiliations:** 1 Department of Molecular Medicine and Surgery, Stockholm Sports Trauma Research Center, Karolinska Institutet, Stockholm, Sweden; 2 Capio Artro Clinic, FIFA Medical Centre of Excellence, Sophiahemmet Hospital, Valhallavägen 91, 11486 Stockholm, Sweden

**Keywords:** ACL, Reoperation, KOOS, PASS, ACL reconstruction, Subjective knee function

## Abstract

**Purpose:**

To analyze minimal important change (MIC), patient-acceptable symptom state (PASS) and treatment failure after reoperation within 2 years of primary ACL reconstruction and compare them with patients without additional surgery.

**Methods:**

This is a retrospective follow-up study of a cohort from a single-clinic database with all primary ACLRs enrolled between 2005 and 2015. Additional surgery within 2 years of the primary ACLR on the ipsilateral knee was identified using procedural codes and analysis of medical records. Patients who completed the Knee injury and Osteoarthritis Outcome Score (KOOS) questionnaire preoperatively and at the 2-year follow-up were included in the study. MIC, PASS and treatment failure thresholds were applied using the aggregate KOOS (KOOS_4_) and the five KOOS subscales.

**Results:**

The cohort included 6030 primary ACLR and from this 1112 (18.4%) subsequent surgeries were performed on 1018 (16.9%) primary ACLRs. 24 months follow-up for KOOS was obtained on 523 patients (54%) in the reoperation group and 2084 (44%) in the no-reoperation group. MIC; the no-reoperation group had a significantly higher improvement on all KOOS subscales, Pain 70.3 vs 60.2% (*p* < 0.01), Symptoms 72.1 vs 57.4% (*p* < 0.01), ADL 56.3 vs 51.2% (*p* < 0.01), Sport/Rec 67.3 vs 54.4% (*p* < 0.01), QoL 73.9 vs 56.3% (*p* < 0.01). PASS; 62% in the non-reoperation group reported their KOOS_4_ scores to be satisfactory, while only 35% reported satisfactory results in the reoperated cohort (*p* < 0.05). Treatment failure; 2% in the non-reoperation group and 6% (*p* < 0.05) in the reoperation group considered their treatment to have failed.

**Conclusion:**

Patients who underwent subsequent surgeries within 2 years of primary ACLR reported significantly inferior outcomes in MIC, PASS and treatment failure compared to the non-reoperated counterpart at the 2-year follow-up. This study provides clinicians with important information and knowledge about the outcomes after an ACLR with subsequent additional surgery.

**Level of evidence:**

III.

## Introduction

Rupture of the anterior cruciate ligament (ACL) is a common injury and the rates of ACL reconstructions (ACLR) among young adults increased in recent years [22, 23, 25]. The primary goal of an ACLR is to restore knee laxity and improve subjective instability. It is widely accepted that, in active patients wishing to return to competitive sports, ACLR is usually recommended [7]. Reoperation rates after primary ACLR vary greatly in the literature and are reported to be between 3.9 and 27.6% [5, 10, 15, 16, 21]. Among the risk factors for subsequent surgery, i.e., both ACL revision and other surgeries are female sex, younger age at the primary ACLR and the use of allograft [2, 15, 20]. However, there are limited data available about reoperations after ACLR, especially regarding non-revision ACLR. Patient-reported outcome measures (PROMs) are generally used as the primary outcome in clinical trials, although in many retrospective ACL cohort studies graft failure is used. The most frequently used PROM is the Knee injury and Osteoarthritis Outcome Score (KOOS) [19]. Most often, PROMs are presented as changes and absolute means but these might be difficult to interpret clinically. To better transfer the data to something that can be interpreted as a clinically relevant improvement or an acceptable result, three definitions are widely used; minimal important change (MIC), patient-acceptable symptom state (PASS) and treatment failure. The MIC is the smallest change in KOOS subscale scores that is considered to be clinically relevant [6]. Ingelsrud et al. [13] recently defined subscale-specific cut-offs for the MIC after an ACLR. While MIC describes a clinically relevant improvement, it can still describe the improvement from bad to mediocre, and not to a satisfactory level of improvement. The PASS answers the question if the patient considers his/her knee function satisfactory and thus tries to identify the patients that consider themselves to be well. Recently, Muller et al. [17] validated the thresholds for the achievement of a PASS for each KOOS subscale. MIC and PASS together complement each other and identify patients feeling better, i.e., achieving the MIC threshold, and feeling good, i.e., achieving the PASS thresholds.

Patient’s perception of failure after an ACLR is as relevant to recognize as a success but it has been a bit neglected in the research. Ingelsrud et al. [12] defined the threshold values for treatment failure for each KOOS subscales considering the patients’ own perception of treatment outcome. In the studies by Cristiani et al. [2] and Barenius et al. [1], almost two-thirds of the patients who underwent ACLR reported a PASS on four of the five KOOS subscales 2 years after surgery, while almost 20% were regarded as treatment failures. While numerous factors, such as age, gender and quadriceps strength affect the accomplishment of a PASS [2] and meniscus surgery is a predictor for treatment failure [1], the influence of subsequent reoperations on the subjective knee outcome after a primary ACLR has not been investigated.

The purpose of this study was to analyse MIC, PASS and treatment failure after subsequent surgeries within 2 years of primary ACL reconstruction and compare them with patients without additional surgery in a large cohort. It is important to know the problems and risks of reoperations that may occur, but knowing the consequences of these is even more important. There are studies that describe reoperations, but none that show the outcome after reoperations. It is important to know to what extent reoperations affect the patient's perceived function and for the surgeon in patient counseling. It was hypothesised that patients with continued problems, which lead to subsequent surgery, would have a poorer subjective outcome after ACLR.

## Materials and methods

Ethical approval for this study was obtained from the regional ethics committee (2016/1613-31/32).

This is a retrospective follow-up study of a cohort from a single-clinic database with all primary ACLRs enrolled between 2005 and 2015. Patient characteristics at baseline and a comparison between the two cohorts are summarised in Table 1. A non-response analysis was made, Table 2. Patients completed the KOOS questionnaire preoperatively and at the 2-year follow-up and those who did not answer the KOOS questionnaire were excluded from the study. During the 2-year follow-up, 146 ACL revisions were made, these patients have been excluded from the study.

**Table 1:** Demographics at primary surgery

	Primary ACLR	Non-reoperation cohort	Reoperation cohort	*p* value*
Number (*n*)	6030	5012	1018	
Age at primary ACLR, years, mean ± SD	28.3 ± 10.7	28.7 ± 10.7	26.1 ± 10.2	< 0.001
Gender (%)				< 0.001
Male	55.5	57.0	48.4	
Female	44.5	43.0	51.6	
Side (%)				n.s
Left	48.6	48.9	47.3	
Right	51.4	51.1	52.7	
Graft type (%)				n.s
HT autograft	93.8	93.7	94.3	
BPTB autograft	6.2	6.3	5.7	
Femoral fixation (%)				0.001
Endobutton	83.5	82.7	87.5	
Rigidfix	11.8	12.5	8.4	
Interference screw	4.6	4.7	4.1	
Other	< 0.1	0.1	0	
Tibial fixation (%)				0.02
AO-screw	75.6	74.9	79.3	
Intrafix	6.3	6.6	4.9	
Interference screw	12.1	12.3	10.9	
Other	6.0	6.2	4.9	
Cartilage injury (%)	18.2	18.5	17.0	n.s
Meniscus injury (%)	47.4	46.4	52.3	0.002
Medial meniscus	23.8	23.6	24.9	
Lateral meniscus	23.6	22.8	27.4	
Medial meniscus resection	14.8	15.2	13.1	n.s
Medial meniscus repair	6.1	5.4	9.2	< 0.001
Lateral meniscus resection	16.2	15.8	18.2	n.s
Lateral meniscus repair	3.9	3.6	5.6	0.003

Data are reported as %, unless otherwise indicated

*ACLR* anterior cruciate ligament reconstruction, *BPTB* bone–patellar tendon–bone, *HT* hamstring tendons, *SD* standard deviation

*p values for comparisons between the non-reoperation and reoperation cohort

**Table 2:** Non-response analysis

	Primary ACLR	Non-reoperation cohort	Reoperation cohort	*p* value*
Number (*n*)	6030	2928	570	
Age at primary ACLR, years, mean ± SD	28.3 ± 10.7	29.4 ± 11.0	25.7 ± 10.0	< 0.001
Gender (%)				n.s
Male	55.5	50.2	46.0	
Female	44.5	49.8	54.0	
Side (%)				n.s
Left	48.6	49.1	50.5	
Right	51.4	50.9	49.5	
Graft type (%)				n.s
HT autograft	93.8	94.6	96.1	
BPTB autograft	6.2	5.4	3.9	
Femoral fixation (%)				0.018
Endobutton	83.5	79.2	85.4	
Rigidfix	11.8	15.6	10.8	
Interference screw	4.6	5.1	3.9	
Other	< 0.1	0.1	0	
Tibial fixation (%)				n.s
AO-screw	75.6	75.1	78.9	
Intrafix	6.3	6.3	5.9	
Interference screw	12.1	13.7	10.1	
Other	6.0	6.9	6.1	
Cartilage injury (%)	18.2	18.3	17.4	n.s
Meniscus injury (%)	47.4	38.0	43.0	0.04
Medial meniscus	23.8	23.4	22.7	
Lateral meniscus	23.6	21.1	27.0	
Medial meniscus resection	14.8	14.8	13.0	n.s
Medial meniscus repair	6.1	4.7	9.3	< 0.001
Lateral meniscus resection	16.2	14.3	17.3	n.s
Lateral meniscus repair	3.9	2.8	3.7	n.s

Data are reported as %, unless otherwise indicated

*ACLR* anterior cruciate ligament reconstruction, *BPTB* bone–patellar tendon–bone, *HT* hamstring tendons, *SD* standard deviation

*p values for comparisons between the non-reoperation and reoperation cohort

Additional surgery within 2 years of the primary ACLR on the ipsilateral knee was identified. Surgical procedures were coded according to NOMESCO (Nordic Medico-Statistical Committee) classification of surgical procedures. If a patient underwent subsequent surgery, medical records were reviewed and the details for the procedure(s) were obtained and classified into 11 different reoperation subgroups (Table 3). For patients with more than one reoperation, for example, septic arthritis that led to more than one reoperation, only the primary reoperation was included in the analysis. Moreover, if more than one procedure was performed during the reoperation, all the procedures were recorded and the same patient can, therefore, be present in more than one subgroup. However, in the comparison between the patients who underwent reoperations and those who did not, one patient cannot be present more than once.

**Table 3:** Reoperation subgroups

Reoperation	Time ACLR-reop (months ± SD)	Number (%)
Screw extraction	12.4 ± 4.2	282 (25.3)
Meniscus procedures	12.5 ± 5.3	238 (21.4)
Notch impingement	11.1 ± 4.9	222 (20)
ACL revision	14.4 ± 5.0	146 (13.1)
Cartilage procedures	13.0 ± 4.3	77 (6.9)
Septic arthritis	0.1 ± 0.3	53 (4.8)
Synovitis	10.6 ± 5.6	31 (2.8)
Diagnostic arthroscopy	12.2 ± 5.5	22 (2.0)
Extraction of loose bodies	13.2 ± 6.8	16 (1.4)
Arthrofibrosis	9.2 ± 5.7	16 (1.4)
Other^a^	13.4 ± 3.6	9 (0.8)
Total	11.1 ± 4.6	1112

*ACL* anterior cruciate ligament

^a^Includes: excision of osteophytes, ganglion or bone, scar correction

The achievement of a PASS and treatment failure on the KOOS was assessed on the basis of the threshold values identified by Muller et al. [17] and by Ingelsrud et al. [17] (Table 4).

**Table 4:** KOOS thresholds

KOOS subscale^a^	MIC	Pass	Treatment failure
Pain	2.5	88.9	57
Symptoms	− 1.2	57.1	56
ADL	2.4	100	71
Sport/rec	12.1	75.0	28
QoL	18.3	62.5	28
KOOS_4_^b^	9	79	42

*KOOS* Knee injury and Osteoarthritis Outcome Score, *MIC* minimal important change, *PASS* patient-acceptable symptom state, *ADL* Activities of Daily Living, *QOL* Knee-Related Quality of Life, *Sport/Rec* Sport and Recreational Function

^a^Range 0–100, worst to best

^b^Scores correspond to the average score for four of the five KOOS subscales: pain, symptoms, sport/recreation and QOL

The scores for MIC were assessed from the studies by Ingelsrud et al. [6, 13] (Table 4). They defined MIC values based on an anchor-based approach, using a predictive model to calculate the improvement for the different subscales on the KOOS.

### Surgical technique

All patients underwent an arthroscopic ACLR with a hamstring tendon (HT) autograft or bone–patellar tendon–bone (BPTB) autograft. The details of ACLR and rehabilitation milestones are previously described in several studies [3, 4, 15]. No major changes were made on the surgical technique during the time period of the study. For the ACLRs performed with HT grafts, the semitendinosus tendon was harvested and prepared as a quadruple graft and if insufficient in length or diameter (< 8 mm), the gracilis tendon was harvested as well. The BPTB graft was harvested as the central third of the patellar tendon with two bone blocks. For femoral fixation, an Endobutton (Smith and Nephew, Andover, Mass, USA) fixation device was routinely used and on the tibial side in the vast majority of cases used Ethibond no. 2 sutures (Ethicon, Sommerville, NJ were tied over a 4.5-mm AO bicortical screw with a washer (Smith and Nephew, Andover, Mass, USA) as a post. In a small minority, rigid fix or interference screw were used for tibial fixation. Meniscal repairs were done with either all inside technique using the FastFix suture anchor device (Smith & Nephew, Andover, Mass, USA) or outside in technique using PDS 0 (Ethicon, Sommerville, NJ, USA), depending on tear location.

All patients underwent a standardized rehabilitation protocol with full weight bearing from the start. If the meniscus was repaired, patients wore a brace with a fixed ROM for a total of 6 weeks, 0°–30° for 2 weeks, 0°–60° for another 2 weeks and 0–90 for the last 2 weeks. Resumption of sport activity at 6 months at the earliest.

### Statistical analysis

Statistical analysis was performed with the SPSS (version 25.0, IBM Corp., NY, USA) software package. Continuous variables were described as the mean (standard deviation (SD)) and categorical variables with count (*n*) and proportions (%). Comparisons between the non-reoperation and reoperation cohort were performed with an independent Student’s *t* test for continuous variables and Pearson’s chi-square test for categorical variables. *p* < 0.05 was considered statistically significant.

## Results

**Fig. 1:**
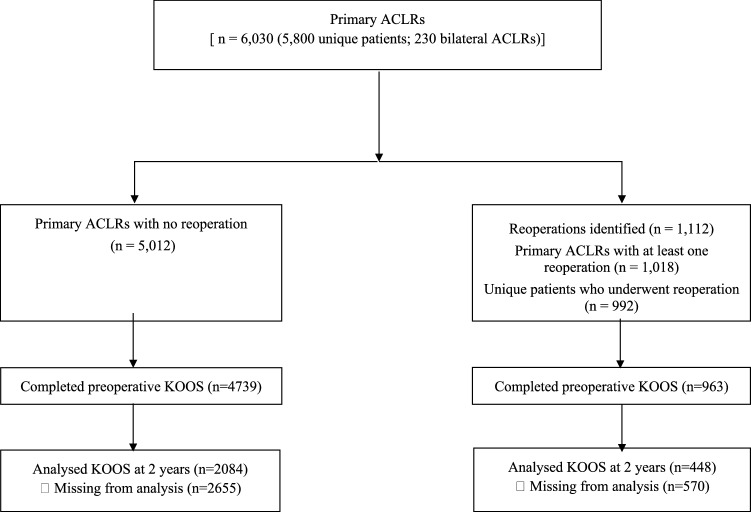
Flowchart. ACLR, anterior cruciate ligament reconstruction *KOOS* Knee injury and Osteoarthritis Outcome Score

A total of 6030 patients who underwent primary ACLR, from 2005 to 2015, were included. From this cohort, 1112 (18.4%) subsequent surgeries were performed on 1018 (16.9%) primary ACLRs, and 992 unique patients underwent reoperation. Two-year follow-up for KOOS was obtained for 448 patients (44%) in the reoperation group and 2084 (44%) in the no-reoperation group (Fig. 1). The mean time to reoperation was 11.1 ± 4.6 months, and the mean time for the different reoperation subgroups is reported in Table 3.

**Fig. 2:**
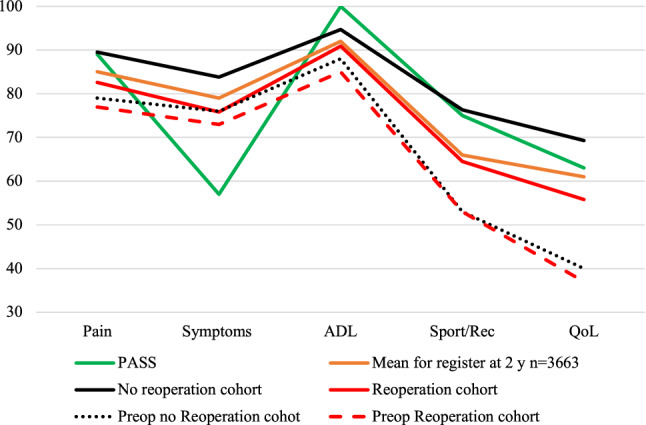
Mean KOOS Scores. *ADL* Activities of Daily Living, *QOL* Knee-Related Quality of Life, *Sport/Rec* Sport and Recreational Function, *PASS* patient-acceptable symptom state, *KOOS* Knee injury and Osteoarthritis Outcome Score

At 2 years, the mean KOOS scores were significantly lower on all subscales for the reoperation group (Fig. 2).

The overwhelming majority of ACLR were done with HT grafts and no difference could be seen between BPTB and HT at risk for reoperations in general, Table 1, due to few patients, we were not able to do further subgroup analysis.

### Minimal important change (MIC)

**Fig. 3:**
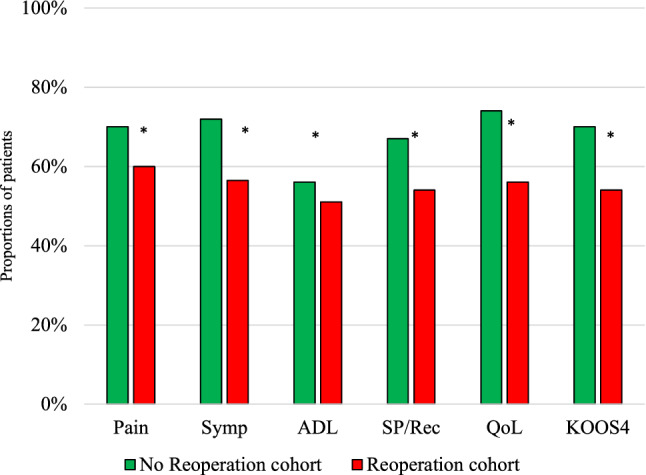
MIC values in the reoperation and non-reoperation cohort. Percentages of patients achieving MIC threshold for each knee injury and Osteoarthritis Outcome Score subscale are given on the *y*-axis. Statistically significant. *p* value < 0.05. *MIC* minimal important change, *ADL* Activities of Daily Living, *QoL* Knee-Related Quality of Life, *Sport/Rec* Sport and Recreation. *Statistically significant

KOOS change from baseline to 2 years showed that the majority of patients reached a minimally important change in improvement. There were, however, significant differences between the two groups on all subscales, Pain 70.3 vs. 60.2% (*p* < 0.01), Symptoms 72.1 vs. 57.4% (*p* < 0.01), ADL 56.3 vs. 51.2% (*p* < 0.01), Sport/Rec 67.3 vs. 54.4% (*p* < 0.01), QoL 73.9 vs.56.3% (*p* < 0.01) (Fig. 3), as well as for KOOS_4_ (Table 5). Patients with subsequent surgeries for notch impingement, arthrofibrosis and extraction of loose bodies had the lowest rate of minimal important change.

**Table 5:** Proportion of patients in the different reoperation groups achieving a PASS and treatment failure

	*n* (%)^d^	Pain	Symptoms	ADL	Sport/Rec	QoL	MIC^a^	PASS^B^	Treatment Failure^c^
No reoperation	2084 (44)	60.7	93.8	46.1	63.0	63.1	70.1	62	2
Reop	448 (44)	40.2	84.5	35.6	34.5	34.7	54.6	35	6
*p* value	< 0.01	< 0.01	< 0.01	< 0.01	< 0.01	< 0.01	< 0.01	< 0.01	< 0.01

*ADL* Activities of Daily Living, *QOL* Knee-Related Quality of Life, *Sport/Rec* Sport and Recreational Function, *PASS* patient-acceptable symptom state

Statistically significant. *p* value < 0.05

^a^MIC according to KOOS_4_

^b^PASS according to KOOS_4_

^c^Treatment failure according to KOOS_4_

^d^Proportion of patients with KOOS 2 years postoperatively

#### PASS

**Fig. 4:**
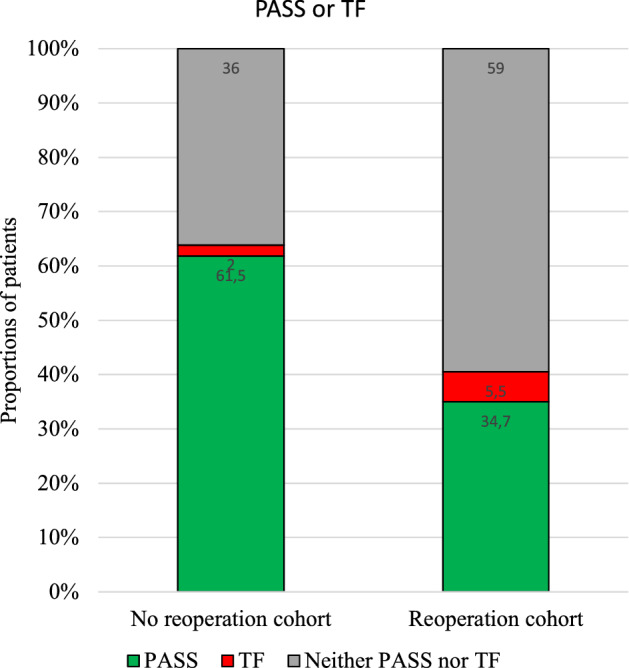
PASS or treatment failure according to KOOS_4_. The percentage of patients with PASS, those with perceived treatment failure, and those belonging to the undecided intermediate group at 2 years after ACLR according to KOOS_4_. *PASS* patient-acceptable symptom state

Applying the PASS thresholds at the 2-year follow-up revealed that 62% of the patients in the non-reoperated group reported their KOOS_4_ scores to be satisfactory, while only 35% reported satisfactory result in the reoperated cohort (Fig. 4).

**Fig. 5:**
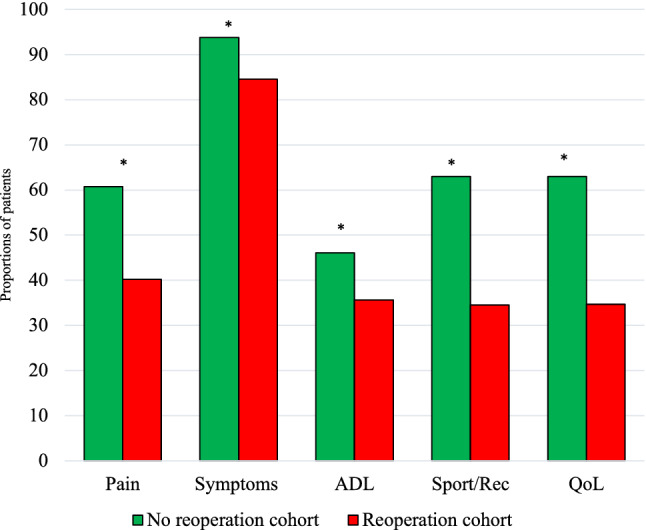
Patient-acceptable symptom state. Percentages of patients achieving PASS for each Knee injury and Osteoarthritis Outcome Score subscale in the reoperation and non-reoperation cohort. Statistically significant. *P* value < 0.05. *ADL* Activities of Daily Living, *QOL* Knee-Related Quality of Life, *Sport/Rec* Sport and Recreation, *PASS* patient-acceptable symptom state. *Statistically significant

The proportion of patients achieving a PASS was significantly lower in all KOOS subscales in the reoperation cohort compared with the non-reoperation cohort (Fig. 5; Table 5). Patients who underwent ACL revision, meniscus procedures or screw extraction achieved a PASS to a larger extent for all KOOS subscales, while patients who underwent subsequent surgery for more undefined problems, such as extraction of loose bodies and diagnostic arthroscopy achieved a PASS to a lower extent (Table 6).

**Table 6:** Proportion of patients in the different reoperation groups achieving a PASS and treatment failure in the KOOS subscales

	*n* (%)^d^	Pain	Symptoms	ADL	Sport/Rec	QoL	MIC^a^	Pass^b^	Treatment failure^c^
Synovitis	19 (63)	42.1	94.7	31.6	26.3	36.8	53.8	21	0
Other	5 (71)	20.0	20.0	20.0	20.0	20.0	50.0	25	25
Screw removal	120 (44)	39.2	89.2	33.3	43.9	42.9	58.8	38	4
Meniscus procedures	130 (68)	43.1	83.8	31.5	42.3	33.6	52.4	36	7
Extraction of loose body	13 (81)	23.1	69.2	15.4	38.5	30.8	30.8	8	15
Diagnostic arthroscopy	10 (50)	40.0	90.0	30.0	30.0	20.0	66.7	20	0
Cartilage procedures	34 (47)	32.4	73.5	17.6	32.3	17.6	44.4	19	10
Arthrofibrosis	5 (42)	20.0	20.0	20.0	33.3	20.0	33.3	33	0
Notch impingement	89 (42)	42.1	73.0	31.6	25.0	33.0	35.5	35	10
Septic arthritis	23 (47)	43.5	87.0	34.8	36.4	40.9	57.9	32	5

*ADL* Activities of Daily Living, *QOL* Knee-Related Quality of Life, *Sport/Rec* Sport and Recreational Function, *PASS* patient-acceptable symptom state

^a^MIC according to KOOS_4_

^b^PASS according to KOOS_4_

^c^Treatment failure according to KOOS_4_

^d^Proportion of patients with KOOS 2 years postoperatively

### Treatment failure

**Fig. 6:**
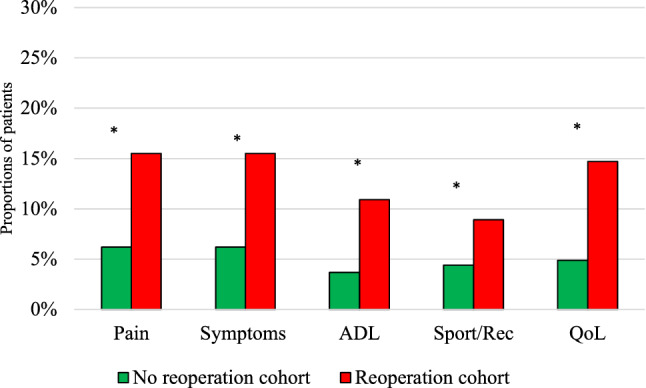
Treatment failure. Percentages of patients with perceived treatment failure for each knee injury and Osteoarthritis Outcome Score subscale in the reoperation and non-reoperation cohort. Statistically significant. *p* value < 0.05. *ADL* Activities of Daily Living, *QOL* Knee-Related Quality of Life, *Sport/Rec* Sport and Recreation. *Statistically significant

When applying the treatment failure thresholds for KOOS_4_, there were significantly differences between the groups; 2% in the non-reoperation group and 6% in the reoperation group (*p* < 0.05) perceived their treatment to have failed (Table 5; Fig. 4). The reoperation cohort had significant more treatment failures in all subgroups compared with the non-reoperation cohort (Fig. 6). In the reoperation subgroups, treatment failure was the highest among those who underwent subsequent surgery due to ‘other’ conditions and extraction of loose bodies (Table 6).

## Discussion

The most important finding in this study was that patients who underwent subsequent surgery within 2 years of a primary ACLR reported significantly inferior outcomes in MIC, PASS and treatment failure compared to the non-reoperated counterpart. In the reoperated cohort, only around 54% felt better (MIC), and only a 35% felt good (PASS) after 2 years, while in the non-reoperated cohort, 70% felt better and almost 62% felt good.

At 2 years, in the reoperated cohort between 9 and 16% perceived themselves as treatment failures, a significant difference compared to the non-reoperated cohort, where only 4–6% considered themselves as treatment failures.

The rates of subsequent surgeries after an ACLR vary between studies. Lord et al. [15] reported that 16.9% of primary ACLRs underwent reoperation within 2 years. Hettrich et al. [11] found reoperation rates of 18.9% at a 6-year follow-up, while Csintalan et al. [5] only found reoperation rate of 3.9% at a mean of 1.9 years. There might be different explanations for the different results between these studies. One could be that some registries have poor coverage and do not register all reoperations. Furthermore, in the study by Csintalan et al., the authors only included the four most common procedures [5]. In the present study, to ensure all subsequent surgeries were reported, medical records from all patients were reviewed and all reoperations performed after the primary ACLR were included.

The outcomes after revision ACLR have been reported in different studies. Several studies evaluating PROMs following ACL revision have demonstrated inferior outcomes compared to primary ACLR [9, 14, 24]. However, there is a lack of literature reporting on outcomes after reoperations for reasons other than revision ACLR.

PROMs provide reports from patients about their functional status, health and quality of life. To interpret the results of the PROMS, several studies have defined MIC, PASS and treatment failure for KOOS subscale scores after ACLR [12, 13, 17]. The evaluation of MIC, PASS and treatment failure has become increasingly important as a method for interpreting patient satisfaction after ACLR. MIC correlates to ‘feeling better’, and most of the patients felt a significant improvement. However, the results of the current study clearly demonstrate that there are significant differences regarding the reoperation and the non-reoperation cohort. The cohort without reoperations had MIC values in line with another study looking at MIC values at 2 years after ACLR [13]. However, when having subsequent surgery after an ACLR, the MIC values were significantly lower on all KOOS subscales, and only roughly half of the patients felt a significant improvement. Applying the PASS thresholds to the patients who underwent a reoperation, a much lower proportion a PASS 2 years after ACLR compared with the non-reoperation cohort (35 vs. 62%). In agreement with our results, Muller et al. [17] reported that patients who did not achieve a PASS had reinjured their knee and underwent subsequent surgery more often than patients who achieved a PASS.

When looking at all the reoperation subgroups, patients who underwent reoperations for more defined problems, i.e., meniscus procedures, ACL revision and screw removal, a larger number of patients achieved the PASS-threshold values, while those with a more undefined problem, such as diagnostic arthroscopy and notch impingement had a higher rate of not achieving a PASS.

The rehabilitation process after cruciate ligament surgery may be stressful mentally and physically. The patient perception of their knee function is associated with the degree of satisfaction after ACLR. It is a complex relationship between subjective outcomes and psychological responses. The question is how much can a person’s mindset interfere with recovery and wellness, or alternatively, compensate for a suboptimal surgical outcome? We have no data on psychological readiness and it is complicated to describe the process leading to the subsequent surgery. One can suppose that for less defined indications psychological factors can be assumed to have a greater impact on outcome but that remains to look into further and is not within the scope of this manuscript. It is important to give the patients information about realistic goals of ACLR to prevent postoperative dissatisfaction and unnecessary reoperations despite a successful operation in the surgeons' point of view. Also, to get past the feeling that surgery has failed when there is a reoperation.

Patients with poor KOOS scores are often overlooked in the literature. Treatment failure has often been assigned by the surgeon or by cut-offs applied without input from patients. Barenius et al. [1] and Frobell et al. [8] both used a KOOS QoL subscale score of < 44 points as a cutoff for treatment failure. This value originated from a study by Frobell et al. [8] the KANON study, in which it was used as a criterion for crossover from nonsurgical to surgical treatment. In Barenius et al. [1] study from the SNKLR, 30% of the patients perceived their treatment as failure, whereas Frobell et al. [8] found 18% treatment failures in their early ACLR group and 27% in the rehabilitation and optional ACLR group. Ingelsrud et al. [12] posed a direct question to patients from the Norwegian Knee Ligament Registry (NKLR) and yielded cut off values for all subscales for treatment failure. In the study, 12% of the patients perceived their treatment as failed 2 years postoperatively. Roos et al. [18] reexamined the KANON cohort with cutoff values originated from the study by Ingelsrud et al. [12] and found that at 2 years almost 10% reported KOOS4 scores below the treatment failure cutoff.

In this study, only 2% of the patients regarded themselves as treatment failure in the cohort without reoperation, whereas in the reoperated cohort 6% according to the KOOS_4_. However, the rates of treatment failure in the different subscales varied between 9 and 16%, which is more in line with other studies [12, 18]. Interestingly, the majority of the patients in the reoperated cohort were unsatisfactory, although did not think that the treatment had failed them. This may be due to a feeling of defeat having had to undergo further surgery, but still having a functional knee.

The main strength of this study was the analysis of a large cohort, with a thorough examination of the material to ensure that all the reoperations were included. Moreover, the study was performed at a specialist knee clinic with a large volume and experience of knee traumatology and ACLR in particular, which may affect the generalizability of the study.

Several limitations are present. Studies from registries suffer in general from loss of follow-up rates, which is the case with this study as well. Furthermore, the results from the present study are only short term, many patients were only assessed within one year from the second surgery. It is a limitation that the same patient can, therefore, be present in more than one subgroup. Another limitation is that this study only comprised patients who underwent both primary ACLR and reoperation at the same high volume surgical clinic. This might affect the generalizability of the data. There is a possibility that some patients underwent subsequent surgery at another clinic. However, patients were actively followed for approximately 12 months after the primary ACLR and were given the possibility to contact the clinic directly in case of postoperative problems or new injuries. There is also the limitation that the reasons for reoperations were interpreted retrospectively from patient’s chart data at time of surgery and no standardized indications for reoperations were present. Finally, in this study, all reoperations were included, making this a heterogenous group with different pathologies, which is a limitation.

## Conclusion

Patients who underwent subsequent surgeries within 2 years of primary ACLR reported significantly inferior outcomes in MIC, PASS and treatment failure compared to the non-reoperated counterpart at the 2-year follow-up. This study provides clinicians with important information and knowledge about the outcomes after an ACLR with eventual additional surgery.
